# Leveraging routine viral load testing to integrate diabetes screening among patients on antiretroviral therapy in Malawi

**DOI:** 10.1093/inthealth/ihaa030

**Published:** 2020-06-17

**Authors:** Victor Singano, Joep J van Oosterhout, Austrida Gondwe, Pearson Nkhoma, Fabian Cataldo, Emmanuel Singogo, Joe Theu, Wilson Ching'ani, Mina C Hosseinpour, Alemayehu Amberbir

**Affiliations:** Dignitas International, Zomba, Malawi; Dignitas International, Zomba, Malawi; Department of Medicine, College of Medicine, Blantyre, Malawi; Dignitas International, Zomba, Malawi; Dignitas International, Zomba, Malawi; Dignitas International, Zomba, Malawi; Dignitas International, Zomba, Malawi; Dignitas International, Zomba, Malawi; Ministry of Health, District Health Office, Zomba, Malawi; University of North Carolina-Malawi Project, Lilongwe, Malawi; Dignitas International, Zomba, Malawi

**Keywords:** diabetes, HIV, integration, screening, viral load

## Abstract

**Background:**

People living with HIV are at an increased risk of diabetes mellitus due to HIV infection and exposure to antiretroviral therapy (ART). Despite this, integrated diabetes screening has not been implemented commonly in African HIV clinics. Our objective was to explore the feasibility of integrating diabetes screening into existing routine HIV viral load (VL) monitoring and to determine a group of HIV patients that benefit from a targeted screening for diabetes.

**Methods:**

A mixed methods study was conducted from January to July 2018 among patients on ART aged≥18 y and healthcare workers at an urban HIV clinic in Zomba Central Hospital, Malawi. Patients who were due for routine VL monitoring underwent a finger-prick for simultaneous point-of-care glucose measurement and dried blood spot sampling for a VL test. Diabetes was diagnosed according to WHO criteria. We collected demographic and medical history information using an interviewer-administered questionnaire and electronic medical records. We conducted focus group discussions among healthcare workers about their experience and perceptions regarding the integrated diabetes screening program.

**Results:**

Of patients undergoing routine VL monitoring, 1316 of 1385 (95%) had simultaneous screening for diabetes during the study period. The median age was 44 y (IQR: 38–53); 61% were female; 28% overweight or obese; and median ART duration was 83 mo (IQR: 48–115). At baseline, median CD4 count was 199 cells/mm^3^ (IQR: 102–277) and 50% were in WHO clinical stages I or II; 45% were previously exposed to stavudine and 88% were virologically suppressed (<1000 copies/mL).  Diabetes prevalence was 31/1316 (2.4%). Diabetes diagnosis was associated with age ≥40 y (adjusted OR [aOR] 7.44; 95% CI: 1.74 to 31.80), being overweight and/or obese (aOR 2.46; 95% CI: 1.13 to 5.38) and being on a protease inhibitor-based ART regimen (aOR 5.78; 95% CI: 2.30 to 14.50). Healthcare workers appreciated integrated diabetes screening but also reported challenges including increased waiting time, additional workload and inadequate communication of results to patients.

**Conclusions:**

Integrating diabetes screening with routine VL monitoring (every 2 y) seems feasible and was valued by healthcare workers. The additional cost of adding diabetes screening into VL clinics requires further study and could benefit from a targeted approach prioritizing patients aged ≥40 y, being overweight/obese and on protease inhibitor-based regimens.

## Introduction

Globally, the burden of diabetes mellitus is rising.^1^ Data from high-income countries show an increased prevalence of diabetes among people living with HIV (PLHIV) on antiretroviral therapy (ART).^2–5^ However, similar studies conducted in sub-Saharan Africa have shown inconsistent prevalence in different study populations of PLHIV on ART.^6^ In Malawi, in a general population survey using the WHO STEPS methodology and another survey in urban and rural Malawi, the prevalence of diabetes in the 15–65 y age group was 5.6 and 3%, respectively,^7,8^ while among patients on ART a varying prevalence of 0.3 to 4.1% was observed.^9,10^

PLHIV on ART are at an increased risk of developing diabetes due to HIV infection, the side effects of ART^11^ and traditional risk factors for diabetes such as increasing age (as a result of prolonged survival on ART), obesity, family history and lifestyle factors (e.g., tobacco and excessive alcohol consumption, physical inactivity and poor diet remain highly prominent drivers of diabetes among PLHIV). Despite the interplay between HIV and ART on diabetes risk, and international recommendations on 6 to 12 mo fasting plasma glucose testing in PLHIV,^12^ screening for diabetes has not been introduced at most ART clinics in Malawi.

Malawi's national HIV program is relatively well developed due to large donor investments and includes free-of-charge treatment provided daily at primary healthcare facilities.^13^ Diabetes care relies primarily on the national health budget; services are not decentralized beyond district hospitals and are affected by regular drug shortages.^14^ In general, HIV and diabetes services in health facilities need to be accessed at separate clinics. Leveraging the capacity of HIV clinics to provide diabetes screening and treatment services enables PLHIV and diabetes to access care during a single visit. PLHIV are already at an increased risk of cardiovascular diseases (CVD)^4^ and undiagnosed diabetes further increases this risk. Integrated diabetes care may contribute to increased survival of patients on ART by improving the management of complications and comorbidities. Integrated care may enhance adherence to treatments, which is essential for successful management of both conditions.^13^ Leveraging HIV services for non-communicable disease (NCD) care may be particularly beneficial in settings with very limited human resources.^13^ As part of a wider initiative to integrate HIV and NCD care at a large HIV clinic in Malawi,^15^ our objective was to assess the feasibility of combining diabetes screening with routine viral load (VL) monitoring and to determine a group of HIV patients who benefit from targeted screening for diabetes.

## Methods

### Study design and setting

We conducted a cross-sectional study at the HIV clinic of Zomba Central Hospital (an urban setting, with about 7000 patients in care) located in the Zomba district, southern Malawi, where HIV prevalence is estimated to be 12.8% in the 15–64 y age group.^16^ We utilized a mixed methods approach with quantitative and qualitative data collection techniques. The study period was from January to July 2018.

### Study participants

**Figure 1.**
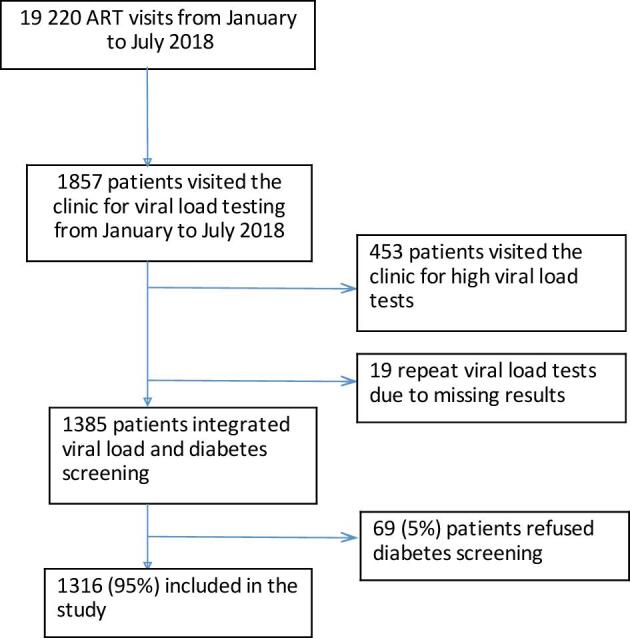
Study flowchart of participants enrolled in the HIV clinic of Zomba Central Hospital.

We enrolled patients on ART aged ≥18 y who visited the ART clinic and were eligible for routine VL testing following ART initiation at 6 mo, 24 mo and 2-y intervals thereafter (Figure 1). Patients were asked if they had been diagnosed with diabetes before and were enrolled to ascertain current glucose levels irrespective of previous diagnosis. Pregnant and breastfeeding women on ART were excluded because they attended a different clinic and because gestational diabetes has a different diagnostic and treatment algorithm. Patients who accessed VL testing for a repeat sample after an initial unsuppressed VL test result (>1000 copies/mL) were also excluded because they would have been captured already during their initial VL test.

### Data collection

We enrolled a convenience sample of consecutive ART patients who underwent routine VL monitoring during the study period. Enrolled clients were interviewed by a trained research assistant using a structured questionnaire for demographic and traditional diabetic risk factors. After the interview, the clients were referred for routine VL testing and blood glucose testing. A trained healthcare worker (an HIV diagnostic assistant^17^) collected a capillary blood sample for a combined blood glucose measurement using a point-of-care SD CHECK GOLD blood glucose meter (SD Biosensor, Inc., GyeongGi-do, Republic of Korea) and for a dried blood spot (DBS) sample for VL testing according to national HIV program guidelines.^18^ DBS samples were transported to the Zomba Central Hospital molecular laboratory for HIV-1 RNA analysis on an (Abbott Laboratories. Abbott Park, Illinois, USA) m2000 real-time system (detection level 40 copies/ml). If the initial random blood glucose (RBG) level was >200 mg/dl, participants were invited for a fasting blood glucose (FBG) test the next morning or as soon as possible. Clients with a second FBG test of >126 mg/dl were referred to a clinician for appropriate management following national NCD guidelines, with integrated follow-up for diabetes and HIV care at the ART clinic.

Additional clinical and HIV care-related information was extracted from electronic medical records including weight and height measurements, current and previous ART regimens, previous exposure to stavudine, duration of ART, history of TB treatment, current TB treatment, VL levels and WHO HIV clinical stage and CD4 count at the start of ART.

Qualitative data were collected through focus group discussions (FGDs) with the use of an interview guide and were audio-recorded. Open-ended questions were asked about the access to, experience with and perceptions about integrated diabetes screening and routine VL monitoring and linkage to diabetes care among HIV clinic healthcare workers. Two FGDs were conducted in English among 15 purposefully selected healthcare workers (two clinicians, five nurses and eight HIV diagnostic assistants).

### Clinical definitions

Diabetes mellitus was defined as an elevated blood glucose level recorded on two separate occasions: RBG >200 mg/dl and FBG >126 mg/dl. Participants with a self-reported diagnosis of diabetes, whether on medications or not, were counted as having diabetes regardless of their blood glucose level.

### Sample size and sampling technique

The study enrolled consecutive patients visiting the routine VL monitoring clinic based on their scheduled appointments for 7 mo. Our expected patient flow of 1300 scheduled clinic visits with a diabetes prevalence of 4.1% among HIV-infected patients from our previous survey^10^ in the clinic provides a precision of ±1% at 95% CI. Healthcare workers working in VL clinics as well as those responsible for managing diabetes and HIV patients were purposefully selected and FGDs were conducted.

### Statistical analysis

Quantitative data were entered in a Microsoft (Microsoft Corporation One Microsoft Way Redmond, WA 98052-6399 USA) Access database, cleaned and analyzed using Stata 13 (StataCorp LP, College Station, TX, USA). We used descriptive statistics to present patients’ demographic and clinical characteristics. Multivariable logistic regression analyses were performed to determine independent associations of demographic and clinical characteristics with diabetes diagnosis. Each participant's age, gender and body mass index (BMI) were included in multivariate analysis as a priori confounders, irrespective of p-values in the univariate analysis, given their known biological importance in relation to diabetes in the literature.^19^ Variables from the univariate analysis with p<0.05 and a priori confounders were fitted in the multivariate analysis. The final model included age, gender, BMI and current ART regimen. The variables with p<0.05 in the multivariate analysis were considered significant.

We developed a thematic framework based on themes that emerged from audio-recorded qualitative data. The main themes were perceptions on and experiences with integrated screening for VL and diabetes and recommendations on how to improve the integration of VL monitoring and diabetes screening. We then coded data based on these themes using NVIVO 11 (QRS International, Burlington, MA 01803, USA).

## Results

### Demographic and clinical characteristics of study participants

A total of 1316/1385 (95%) of HIV-positive participants undergoing routine VL monitoring were simultaneously screened for diabetes during the study period. The diabetes prevalence was 2.4% (31/1316), of whom 2/31 (6.5%) were newly diagnosed diabetes cases, both linked to integrated diabetes care. Of the patients simultaneously screened, the median age was 44 y (IQR: 38–53); 61% were female; and 28% were overweight or obese. At the start of ART, the median CD4 count was 199 cells/mm^3^ (IQR: 102–277) and 50% were in WHO clinical stages I or II. The median ART duration was 83 mo (IQR: 48–115) and the prevalence of previous exposure to stavudine was 45%. The majority of participants (82%) were on a fixed-drug combination tenofovir, lamivudine and efavirenz regimen while 6% were on a protease inhibitor-based regimen. Overall, 88% were virologically suppressed (Table 1).

**Table 1.** Demographic and clinical characteristics of participants

Characteristic	Number (%)
*Participants enrolled (N=1316)*	1316
*Diabetes cases (n=31)*	31 (2.4)
Newly diagnosed	2 (6.5)
Previously diagnosed	29 (93.5)
*Age, y (N=1316)*	
Median age (IQR)	44 (38–53)
18–29	89 (6.8)
30–39	336 (25.5)
40–49	450 (34.2)
≥50	441 (33.5)
*Gender (N=1316)*	
Male	509 (38.7)
Female	807 (61.3)
*BMI, kg/m^2^ (N=1316)*	
Median BMI (IQR)	22.2 (20–25)
Underweight	126 (9.6)
Normal	828 (63.0)
Overweight	249 (19.0)
Obese	113 (8.6)
*CD4 count at ART initiation, cells/mm^3^ (N=1316)*	
Median CD4 (IQR)	199 (102–277)
<250	410 (31.2)
≥250	203 (15.4)
Missing	703 (53.4)
*Viral load (N=1316)*	
<1000 copies/ml	1,163 (88.4)
>1000 copies/ml	103 (7.8)
Missing	50 (3.8)
*WHO disease stage at ART initiation (N=1316)*	
Stage I	399 (30.3)
Stage II	259 (19.7)
Stage III	290 (22.0)
Stage IV	89 (6.8)
Missing	279 (21.2)
*Current smoking status (N=1316)*	
Yes	47 (3.6)
No	1269 (96.4)
*Current alcohol use status (N=1316)*	
Yes	137 (10.4)
No	1,179 (89.6)
Current ART regimen* *(N=1316)*	
AZT/3TC/NVP	105 (8.0)
TDF/3TC/EFV	1,075 (81.7)
TDF/3TC + NVP	53 (4.0)
AZT/3TC + ATV/r and TDF/3TC + ATV/r	80 (6.0)
Non-standard regimen	3 (0.2)
*ART duration (N=1305)*	
<24 mo	193 (14.8)
≥24 mo	1,112 (85.2)
Median ART duration, mo (IQR)	82.9 (48.6–115.1)
*Current TB treatment (N=1316)*	
Yes	8 (0.6)
No	1,308 (99.4)
*Previous exposure to stavudine (%) (N=1285)*	
No	662 (50.3)
Yes	585 (44.5)
Missing	69 (5.2)

BMI, body mass index

* Current ART regimen descriptions: AZT/3TC/NVP = fixed-dose combination of zidovudine, lamivudine and niverapine; TDF/3TC/EFV = fixed-dose combination of tenofovir, lamivudine and efavirens; TDF/3TC + NVP = combination dose of tenofovir, lamivudine and niverapine; AZT/3TC + ATV/r = combination dose of zidovudine, lamivudine and atazanavir/ritonavir; DF/3TC + ATV/r = combination dose of tenofovir, lamivudine and atazanavir/ritonavir.

### Factors associated with diabetes

In the univariable and multivariable analyses, age ≥40 y (adjusted OR [aOR]: 7.44; 95% CI 1.74 to 31.80; p=0.01), being overweight and/or obese (aOR: 2.46; 95% CI 1.13 to 5.38; p=0.02) and being on a protease inhibitor-based regimen (aOR: 5.78; 95% CI 2.30 to 14.50; p<0.01) were significantly associated with a diagnosis of diabetes. There was no significant association with gender, smoking, alcohol use, previous exposure to stavudine, WHO HIV disease stage and CD4 count at ART initiation, current VL, current TB treatment and duration on ART (Table 2).

**Table 2.** Factors associated with diabetes diagnosis among patients on ART at the Zomba Central Hospital HIV clinic

	Diabetes					
Characteristic	Cases with diabetes, n (%)	Cases without diabetes, n (%)	Crude OR (95% CI)	p-value	Adjusted OR (95% CI)*	p-value
*Age, y (N=1316)*				0.00		**0.01**
18–39	2 (0.5)	423 (99.5)	Ref		**Ref**	
≥40	29 (3.3)	862 (96.8)	7.12 (1.69 to 30.00)		**7.44 (1.74 to 31.80)**	
*Gender (N=1316)*				NS		NS^¥^
Male	12 (2.4)	497 (97.6)	Ref		Ref	
Female	19 (2.4)	788 (97.7)	1.00 (0.48 to 2.08)		0.98 (0.45 to 2.10)	
*BMI, kg/m^2^ (N=1316)*				NS		NS
Underweight	3 (3.4)	123 (97.6)	1.42 (0.40 to 5.01)		1.45 (0.40 to 5.18)	NS
Normal	14 (1.7)	814 (98.3)	Ref		**Ref**	
Overweight/Obesity	14 (3.9)	348 (96.1)	2.34 (1.10 to 4.96)		**2.46 (1.13 to 5.38)**	**0.02**
*CD4 count at ART initiation, cells/mm^3^(N=613)*				NS		
CD4 <250	10 (2.4)	400 (97.6)	Ref			
CD4 ≥250	7 (3.5)	196 (96.6)	1.43 (0.54 to 3.81)			
*Viral load (N=1266)*				NS		
<1000 copies/ml	20 (1.7)	1143 (98.3)	Ref			
>1000 copies/ml	2 (1.9)	101 (98.1)	1.13 (0.26 to 4.91)			
*WHO disease stage at ART initiation (N=1037)*				NS		
Stage I/II	14 (2.1)	644 (97.9)	Ref			
Stage III/IV	11 (2.9)	368 (97.1)	1.38 (0.62 to 3.06)			
*Smoking status (N=1316)*						
Yes	0 (0.0)	47 (100,0)	-			
No	31 (2.4)	1238 (97.6)	-			
*Current alcohol use status (N=1316)*						
Yes	0 (0.0)	137 (100.0)	-			
No	31 (2.6)	1148 (97.4)	-			
*Current ART regimen (N=1313)*				0.01		**0.01** ^¥^
zidovudine, lamivudine, nevirapine	3 (2.9)	102 (97.1)	1.63 (0.48 to 5.62)		1.29 (0.37 to 4.48)	NS
tenofovir, lamivudine, efavirenz	19 (1.8)	1056 (98.2)	Ref		Ref	
tenofovir, lamivudine and nevirapine	2 (3.8)	51 (96.2)	2.18 (0.49 to 9.61)		1.94 (0.43 to 8.70)	NS
PI-based regimens*	7 (8.8)	73 (91.3)	5.33 (2.17 to 13.08)		**5.78 (2.30 to 14.50)**	**<0.01**
*Current TB treatment (N=1316)*						
Yes	0 (0.0)	8 (100.0)	-			
No	31 (2.4)	1277 (97.6)	-			
*ART duration (N=1306)*				NS		
<24 mo	3 (1.6)	190 (98.5)	1			
≥24 mo	27 (2.4)	1085 (97.6)	1.58 (0.47 to 5.25)			
*Previous exposure to stavudine (N=1285)*				NS		
No	9 (1.4)	579 (99.0)	1			
Yes	6 1.0)	653 (98.6)	0.75 (0.27 to 2.13)			

NS, non-significant p-value.

*Protease inhibitor (PI)-based regimens: tenofovir, lamivudine and atazanavir/ritonavir; zidovudine, lamivudine and atazanavir/ritonavir.

**The final model included age, gender, body mass index and current ART regimen. Gender and body mass index (BMI) were included in the model as a priori regardless of significance.

¥ Likelihood ratio test (LHR test).

### Facilitators to access, experience and perceptions of integrated diabetes screening and routine VL testing

Two FGDs were conducted among 15 healthcare workers (eight HIV diagnostic assistants, two clinicians and five nurses), of whom eight were male and seven female; their median age was 35 y (IQR: 28–39).

### Saving time and reduction of hospital visits

The integration of diabetes and ART services has reduced the number of hospital visits and transportation costs for patients. Healthcare workers mentioned that it enabled access to both diabetes and ART care during one visit:

*There is an advantage because this program has helped to reduce hospital visits that clients take as they get two different treatments or help in just one day* (HIV diagnostic assistant).

It is therefore noted that conducting diabetes screening and VL monitoring at once saves time for both patients and healthcare workers:

*On the part of us, the HIV diagnostic assistants, it has helped us save time because we conducted both tests at the same time… and identify the normal and abnormal cases right away* (HIV diagnostic assistant).

### Awareness of one's diabetes status

Furthermore, the integration of diabetes screening and routine VL monitoring has contributed to the awareness of diabetes status for patients on ART:

*After some time we have generally realized that this program is a good thing, that is, we have known that some ART drugs can cause diabetes. In the past we did not know that some of our clients have diabetes but now we have known that there is some proportion of our clients that are actually diabetic and not only finding that we have this proportion but also generally improving their care* (nurse).

*If someone has been diagnosed as diabetic earlier, it is good because then it means he/she will be helped earlier than it would have been if we waited for the client to fall sick first before being diagnosed with high blood sugar* (clinician).

*If the client's blood glucose is high, we refer them to the clinicians so that they are guided on what to do… because one may start medication before things get worse* (HIV diagnostic assistant).

### Barriers to integrated diabetes screening routine VL testing and linkage to diabetes care

Healthcare workers noted shortages of diabetes testing kits and drugs at the HIV clinic:

*Test kits are in short supply. We only have one glucometer and it becomes a challenge when we have a lot of clients waiting to be assisted and the glucometer has to be shared among three HIV diagnostic assistants. So because of this, we let other clients go home with a promise that their blood sugar will be tested when they next visit the clinic* (HIV diagnostic assistant).

### Increased workload due to the integrated blood sugar screening and VL testing

The integration of diabetes screening and VL monitoring was coupled with integrated diabetes and HIV treatment for those with comorbidities. This has increased the workload for healthcare providers; previously they were just providing ART but now they also have to provide diabetes care to ART patients who are diabetic:

*I would say, as* [a] *clinician, this program might increase our workload, ideally it will improve the patient's health but at the same time if these cases are many then it will mean our workload is huge because we will have to deal first with their HIV treatment then the person's diabetes treatment* (clinician).

*We only receive clients whose glucose levels are really high, that is above 200 mg/dl either random or fasting blood glucose, those below 200 mg/dl are left to go home so in terms of referring, I think it is ok but the challenge comes in when they come to clinicians because it means we have to conduct counseling on diet and provide lifestyle advice. For one to be properly counseled, it needs about 30 minutes but we do not have time because of other clients waiting outside, therefore the counseling is poor. When the patient has come for treatment, the challenge comes in because they need to be on a queue, so they get the help they need and because of this some do just decide to go home and not take their drugs because the queue is too long and they are tired* (clinician).

### Inadequate feedback to patients on diabetes screening outcomes

There is limited interest among some HIV clinic staff regarding providing diabetes screening. Healthcare providers may concentrate more on HIV than diabetes. As a result, they may miss a diabetes diagnosis at the ART clinic:

*Those* [HIV diagnostic assistants] *conducting the test are not able to interpret the results but clinicians are. Therefore, this is a challenge because the HIV diagnostic assistants only concentrate on viral load and not on diabetes screening. Those that have been on diabetes treatment for some time are the ones that send information to the newly diagnosed on where to get treatment and care* (clinician).

## Discussion

We developed a new approach of combined VL testing and diabetes screening and studied its feasibility in a routine ART clinic implementation setting. We have demonstrated that diabetes screening can be integrated into the routine VL monitoring schedule at a busy, low-resourced, programmatic setting in Malawi. This was highlighted by the high uptake of diabetes screening (95%) and the generally positive perceptions of healthcare workers about the benefits of an early diabetes diagnosis and easy access to diabetes care. We found a modest prevalence of diabetes among patients on ART (2.4%). Diabetes diagnosis was associated with well-recognized risk factors: increasing age, overweight/obesity and being on a protease inhibitor-based regimen, suggesting potential avenues to increase the yield from diabetes screening in this population.

The low prevalence of diabetes in our population on ART is comparable with studies from this region (2–6%) of the general population^7,8^ and of patients on ART.^10,20,21^ While diabetes is associated with vascular morbidity and mortality, and this is important in populations on ART who live longer with chronic morbidities and have increased CVD risk, the low diabetes prevalence does not seem to justify screening all ART patients annually, given the current annual frequency of routine VL testing recommended by the WHO and implemented in Malawi since 2018. Moreover, the cost of diagnostic tests, space availability, supply chain for the reagents and inadequate human resources hamper routine screening for diabetes for patients starting or currently on ART.^22^ In addition, healthcare worker training is time- and resource-intensive, particularly during the initial phase of the program. Follow-up mentoring and feedback may also be needed and staff turnover is a significant challenge.^23^ In an HIV setting, using existing staff who are conducting HIV and VL tests would be vital but the supply chain challenges require close monitoring.

It seems reasonable and more likely to be cost-effective to select higher-risk patients, who are older, overweight and/or obese and are on a protease inhibitor-based ART regimen. Diabetes has been associated with exposure to stavudine, zidovudine and protease inhibitor-based regimens due to altered adiposity, dyslipidemias and insulin resistance, even although its incidence over time has declined with the scaling up of recent ART regimens in the general population.^11^ In this study, only protease inhibitor-based regimens were associated with diabetes but not exposure to zidovudine and stavudine, which may be due to the small numbers of patients with diabetes exposed to these regimens. Whether excessive weight gain on dolutegravir-based regimens, observed in recent African studies, translates into increased diabetes risk, is currently unknown.^24^

From October 2015, we implemented a model of integrated HIV-NCD care at the large HIV clinic at Zomba Central Hospital in southeast Malawi. Initially we referred all patients for diabetes screening regardless of the VL schedule, but this overwhelmed the HIV clinic testing cadres.^15^ We then introduced RBG screening every 2 y in line with the national VL testing schedule. A high uptake of integrated screening for diabetes and VL was possible due to rearrangement of the patient flow, which allowed for efficient finger-prick capillary sampling for point-of-care blood glucose and VL DBS in a single room offered by a single person. Infrastructure modification and patient flow assessments are a critical component of successful integrated NCD-HIV clinical care models.^25^ In our setting, no additional human resources were required to conduct diabetes screening. The study leveraged available HIV testing staff (HIV diagnostic assistants) to conduct simultaneous diabetes and VL tests. While diabetes screening was easily embedded, the extra human resource burden resulting from integrated diabetes treatment was cited as a concern for integrated HIV-NCD programs, despite a low prevalence of diabetes.^26^

Healthcare workers in our study found that the integrated screening model increased the awareness of diabetes and were satisfied with the integrated screening as they recognized it as a point of entry into (integrated) diabetes care. However, focus group discussants mentioned some concerns about integrated diabetes screening increasing the workload for clinicians, due to increasing the burden of integrated diabetes care. In Swaziland, screening for hypertension and diabetes among patients on ART increased the total visit time from a median of 4 to 15 min, even although the time spent on HIV care was not statistically significant.^27^ Increased workload was also mentioned in another integrated HIV chronic care clinic model in Malawi.^15,25^ In our own experience, integrated screening and treatment for hypertension and diabetes increased the mean duration of ART visit time for clinical officers during the first quarter, but returned to baseline thereafter.^15^ Patients attending an integrated NCD-HIV clinic in Swaziland expressed willingness to spend at least 10 min extra for annual screening for hypertension and diabetes.^27^ Further information about how ART patients regard integrated HIV-NCD screening and care is limited.

The strengths of the study include the use of real-life data from a routine programmatic setting, a large sample size, high response rate and the use of mixed quantitative and qualitative methods. Our study also has some important limitations. The single clinic experience limits extrapolation to other settings. Our study excluded pregnant women and findings do not relate to this group. The cross-sectional methodology precludes conclusions about associations being causative. We were unable to collect qualitative data from patients in ART care and therefore lack insight into their knowledge, attitudes and perceptions concerning integrated VL testing-diabetes screening.

### Conclusions

Our innovation of integrating screening for diabetes with routine VL monitoring was feasible, had a very high uptake and was appreciated by healthcare workers at the ART clinic. In the light of low diabetes prevalence, diabetes screening for adults may be prioritized for those at increased risk (aged ≥40 y, overweight/obese, on protease inhibitor-based ART regimens). Further implementation research is needed to determine the cost-effectiveness of integrated diabetes screening and for the consequences of an increased burden of integrated HIV-diabetes care.

## Data Availability

The datasets used and/or analyzed during the current study will be available from the corresponding author on reasonable request.
